# Compendium of Safety Regulatory for Safe Applications of Aerogels

**DOI:** 10.3390/gels9110842

**Published:** 2023-10-24

**Authors:** Antonella Caterina Boccia, Alfio Pulvirenti, Carlos A. García-González, Fabia Grisi, Monica Neagu

**Affiliations:** 1 CNR National Research Council, Istituto di Scienze e Tecnologie Chimiche-SCITEC “G. Natta”, Via A. Corti, 12, 20133 Milan, Italy; alfio.pulvirenti@scitec.cnr.it; 2 AerogelsLab, I+D Farma Group (GI 1645), Departament of Pharmacology, Pharmacy and Pharmaceutical Technology, Faculty of Pharmacy, iMATUS and Health Research Institute of Santiago de Compostela (IDIS), Universidade de Santiago de Compostela, E-15782 Santiago de Compostela, Spain; carlos.garcia@usc.es; 3 Dipartimento di Chimica e Biologia “A. Zambelli”, and INSTM Research Unit, Università di Salerno, Via Giovanni Paolo II 132, 84084 Fisciano, Italy; fgrisi@unisa.it; 4 Victor Babes National Institute of Pathology, 050096 Bucharest, Romania; neagu.monica@gmail.com; 5 Colentina Clinical Hospital, 020125 Bucharest, Romania

**Keywords:** best practices, safety regulatory compendium, aerogels

## Abstract

An increasing number of aerogels as nanostructured highly porous materials are entering the market in every day products, with an attractive portfolio of properties for emerging applications ranging from health care and leisure to electronics, cosmetics, energy, agriculture, food and environmental. However, the novelty in properties and forms of aerogels makes the development of a legislative framework particularly challenging for ensuring the safe development and use of nano-enabled products. The presented safety regulatory Compendium intends to share knowledge with the international aerogels community, as well as end-users and stakeholders, on the regulatory and safe handling procedures, as best safety practices, to be followed during the production process, handling, transport and end-use of aerogel-based formulations to mitigate human and environmental risks considering lack of data availability for this purpose in general.

## 1. Introduction

**Figure 1:**
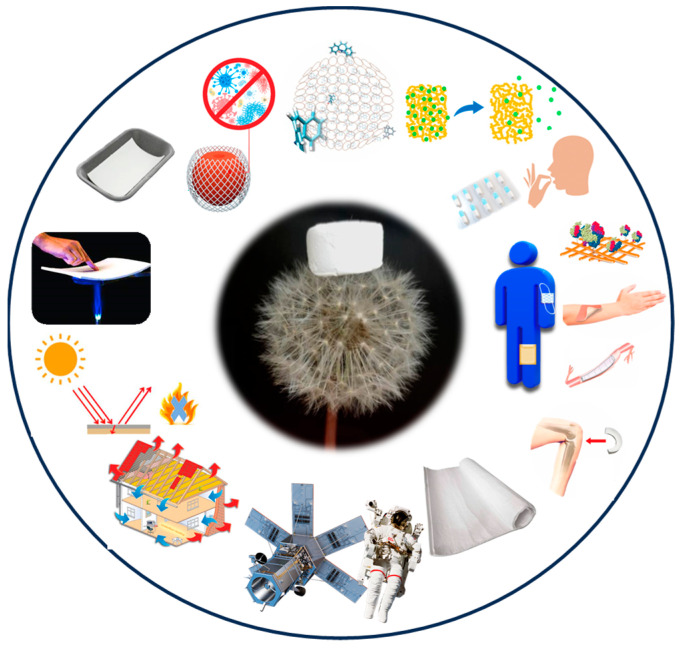
Aerogel-based materials for biomedical, textile, packaging, energy storage devices, solar-steam generation, and aerospace engineering applications.

Aerogels are a special class of nanostructured materials generally with an ultra-light weight and high porosity, associated with tunable physicochemical properties [1]. Kistler reported, for the first time, the term aerogel in 1931, defining it as a low-density, porous solid gel derived from gel [2]. To date, the definition of aerogels lies in a sort of “limbo”, considering that IUPAC defines aerogels as “a gel comprised of microporous solid in which the dispersed phase is gas” [3], but the scientific community stated that aerogels are not strictly microporous solids as there are commonly reported mesoporous and nanofibril aerogels [4]. In the meantime, aerogels cannot be considered nanoforms, based on the REACH Annexes definition [5,6], and they are also exempted from the need to report to national nanomaterials product inventories such as those in France, Belgium, the USA or Canada [7,8]; but, they can be included in the nanomaterials category considering that they are constituted by nanomaterials [9]. Typically, aerogels are obtained through a sol-gel process [10], meaning that a colloidal suspension of precursors was converted by applying a gelling process, after which the replacement of the solvent with gas into materials characterized by a three-dimensional network follows. According to the nature of the solvent occupying the solid scaffold, as well as on the procedure commonly used for the solvent replacement, it is possible to produce: (i) aerogels (usually obtained by CO_2_ supercritical drying) [2,11]; (ii) xerogels (usually obtained by atmospheric pressure drying) [12,13]; and (iii) cryogels (usually obtained by freeze drying) [14], which are materials characterized by different properties and are accepted with the general term “aerogel”. The main sources for fabricating the aerogels are silica, alumina and carbon, but synthetic polymers, biopolymers and other organic precursors need to be considered as emerging sources. In recent years, aerogels are increasingly being studied considering the appealing applications in food, for the controlled release of active compounds, biomedical, textile, packaging, energy storage devices, aerospace engineering, and solar-steam generation [15,16,17], as illustrated in Figure 1, but the full potential of aerogels is still to be assessed for other technological sectors [18,19]. As materials for biomedical applications, they are able to respond to the requirement for tissue engineering applications, such as in designing implantable cardiovascular devices, as well as for nerve repair implant applications, bone grafting and biosensing [20]. The adaptable surface area, together with the surface functionalization, makes them valuable candidates to be used as a platform for adsorbing and controlling the release of active compounds. For specific administration routes, aerogels are capable to improve the delivery of low water soluble drugs and deliver, in situ, the drug enhancing the bioavailability [21]. Concerning aerogels in textiles, they received attention in this century for producing protective clothing for space exploration, as the first application, and successively for thermal insulation textiles [22]. Recently, the new emerging applications include aerogels as textiles for wound care medical applications [23,24], face masks [25] and tissue engineering applications [26,27], thermal insulating [28], smart clothes, prevention of electromagnetic radiation, protection against chemicals, flame retardancy and treatment of textile process wastes [29,30]. Regarding the environmental applications, aerogels are ideal materials for thermal insulations which can save energy and help to reduce carbon emissions due to their low thermal conductivity. Another aspect to be considered is that aerogels generally possess a high porosity and specific surface area, enabling them to be ideal materials useful to adsorb toxic molecules or ions in the air and water, which can be applied to protect the environment [31]. As a consequence, the aerogels market has recently seen a surge in the number of patents filed on aerogels, demonstrating the incredible increase on these materials in recent years. Even considering that the market is mainly driven by the increasing demand for construction applications, the new emerging trend, together with increases in R&D investment and a global aerogel market size projected to reach USD 1045 million by 2025, suggests that the market believes aerogels will become increasingly important [32,33]. 

To date, there is a lack of information concerning the toxicity of aerogels; ecotoxicity and cytotoxicity and regarding the health risk assessment and other regulatory aspects are not extensively addressed as aerogels do not require registration as nanoforms but their nanostructures raise concerns about a possible hazard assessment which needs to be addressed. The reason for this may be partially due to an uncertainty about which aspects should be approached for regulatory purposes. However, producers cannot neglect that even if the toxicity inherent to aerogel exposure is not expected in general, an increased bioactivity may derive from inhalable or ingestible fragments due to their high inner surface area [34]. One of the most critical routes of exposure to aerogel nanoparticles is the unintended inhalation of material dust and the consequent pulmonary deposition, which is an existing scenario in the industrial insulation production of silica and PU-based (PU = polyurethanes) aerogel materials [35], as well as during the installation and removal activities of insulation materials in houses, considering that this is one of the most frequent applications. It is important to keep in mind that for the industrial implementation of aerogels all workers involved in the production process and application of aerogels may be involved in the exposure to these nanostructures and the dispersion into the surrounding environment, and, consequently, global regulation is highly necessary to prevent any risks to human health.

**Figure 2:**
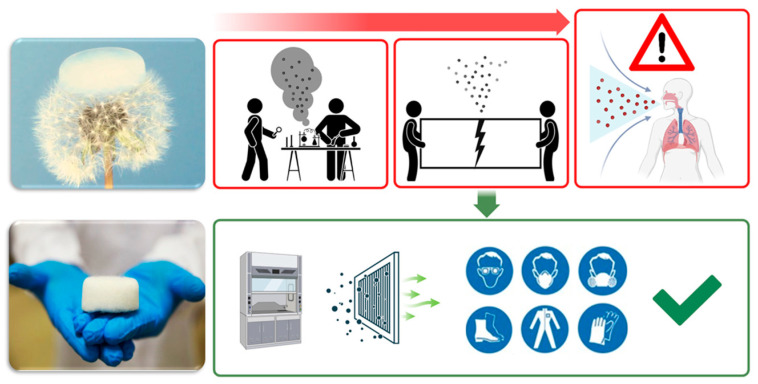
Compendium perspectives. In the green box are illustrated some protection measures to be adopted to limit risks from aerogel particle exposure.

The present work aims to collect and analyze the existing workplace safety regulations that are mainly based on silica aerogel materials for the purpose of evidencing the pressing demand concerning the protection of fundamental rights or risks to human health deriving from the lack of stringent regulation. In this perspective, this compendium aspires to promote to the diffusion of knowledge of security and safety standards, as a guideline of best practices, to be followed during the production process, handling, transport and end-use of aerogel-based formulations that rely on the treatment of nanomaterials to further mitigate potential human health risks (Figure 2) [36,37]. 

Furthermore, it is important to underline that appropriate safety rules must be also extended and applied to all the chemicals involved in the manufacturing of aerogel products, identifying hazards according to EC-Directives 67/548/EC, 1999/45/EC, their various amendments and adaptations, and EC-Regulation 1272/2008 (CLP) [38], all of which must be aligned with current European legislation: REACH (Registration, Evaluation, Authorization and Restriction of Chemicals) [39,40,41], ECHA European Chemicals Agency) [42], Globally Harmonized System (GHS) [43] and other organizations, and together with the existing protocols that can be used. Specifically, the aerogel’s composition, i.e., the information on the ingredients from with it is made, has to be classified as “non-hazardous” in accordance to Regulation (EC) 1272/2008—Annex VI [38], as amended by Regulation (EC) 790/2009 according to CLP criteria [40]. Considering this, in addition to the legislative aspects, a selection of adequate personal protective equipment and indoor air cleaning devices for use during aerogel production and handling are proposed as landmarks for workplace safety regulations and safe handling practices [44].

## 2. Methodology

### 2.1. Safe Handling Precautions

Generally, exposure to aerogels in the workplace can occur during all of the handling related to the manufacturing process. Even if the aerogels are in the form of panels, sheets or similar, it is necessary to consider that they can release powder of an amorphous and inert nature when mechanically stressed, hence releasing particles of different sizes, from inhalable (2.5–10 μm of diameter) to ultrafine particles (less then 0.1 μm of diameter). Therefore, the following recommendations should be observed during aerogel handling, processing and installation to minimize the exposure [44,45]:

- Keep the product in its packaging until the moment it is used;
- Remove the product from its packaging in the working area to limit the area of possible dust diffusion;
- Avoid dust cloud formation;
- Handle and cut in open-air areas or in aerated environments, adequately distanced from other people. Avoid contamination of places and people not involved in the activity;
- Common workplace hygiene practices should be adopted for dust exposure handling. Local exhaust ventilation (LEV) should be the main method of dust control in the working environment. Therefore, adequate ventilation of the environment should be guaranteed to reduce dust concentration.
- Waste products must be stored for disposal in closed bags for reducing dispersion of dust in the working environment;
- In the case of unintentional dispersion, the most effective method of collecting dust is dry vacuuming with a commercial HEPA filter as a better way for dust cleaning compared to sweeping or cleaning with water [46].

### 2.2. Personal Protection Measures 

Personal protective equipment (PPE) shall be consistent with good industrial hygiene and safety practices to avoid respiratory and skin irritation [47]. The referred to protective equipment and specific garments to be adopted for limiting aerogels exposure are as follows.

**Respiratory system protection**: During handling, processing and installation, the use of certified FFP2 grade disposable masks which are CE approved should be mandatory and shall be worn adherent to the face.

**Protection of Hands and other exposed parts of the body** (for skin protection): To prevent any irritation, waterproof gloves, long sleeved clothing and long trousers or, alternatively, full-length disposable overalls should be worn. When one or more substances are handled, the requirement is that the hazard has to be clearly specified, thus enabling workers to properly identify the type of gloves to be worn for avoiding potential contact and with regard to the amount and duration of dermal exposure. Details including the material composition of gloves, the thickness and the typical or minimum breakthrough time should be easily available. In cases of no specific information, the use of impermeable inner gloves and/or cut-resistant outer gloves is recommended. Protective clothing which is able to limit the risk exposure to aeroparticles for workers is governed by CEN/TR 15419 [48] and EN 1073 [49] standards with the intent of assisting users and specifiers in selecting the correct type of protective clothing for the task to be performed and to help them ensure it is used according to the manufacturer’s instructions to provide adequate protection during its entire lifetime.

**Eye protection**: It is recommended to use protective glasses with side screens, specifically, for dust protection.

**Other**: Emergency eyewash and a safety shower should be located nearby.

### 2.3. Exposure Limits and Control

Currently, there is limited nano-specific exposure information in the registration dossiers submitted to ECHA, and no exposure limits have been identified for these products. Workplace exposure limits for the components of the aerogels should be provided by the specific legislature of the workplace’s country for ensuring the application of health surveillance and the safeguarding of workers, as stated by the law. If not available, a guideline, but not an exhaustive indicator, should refer to 8 h exposure (TWA) for silica-based nanoparticles [50,51,52]. For synthetic amorphous silica, the limits are US OSHA PEL (TWA), 10 mg/m^3^ total—5 mg/m^3^ respirable fraction and UK WEL, 6 mg/m^3^ total inhalable—5 mg/m^3^ respirable fraction [47]. The regulatory exposure limits refer to amorphous silica and are intended for use with the general terms of silica, CAS RN 7631-86-9 [53], as stated in Table 1.

In occupational settings, evidence of technical measurement difficulties related to background nanoaerosols has been reported in several studies (ECHA Guidance, Appendix to Chapter R.14, 2012). The ECHA acknowledges that measuring NM exposure is a complicated task, considering the difficulty in measuring the concentration of nanoparticles and evaluating the differentiation between indoor or outdoor activities involving nanoparticles; no single approach can currently be used or recommended given that the most appropriate choice depends on substance-specific information and the measuring techniques available. For instance, the availability and type of hazard information (PNEC/DNEL/OEL and metrics) and the ability to measure PNEC/DNEL/OEL levels (and metric(s)) [54]. Aerogel-based materials and products can develop dust during all steps regarding production, handling, cutting, transport and deposition, and the released particles can have a diameter in the 2.5 and 10 μm range so they are considered inhalable particles. Moreover, an aeroparticle’s diameter should be smaller than 2.5 μm; thus, they are able to penetrate alveoli. Even a diameter smaller than 0.1 μm should be considered because they are known to be capable of reaching the cardiovascular system. Guidelines establishing how to monitor and measure the concentration in the air of nanoparticles are reported from the International Organization for Standardization (ISO) as ISO/TR 12885 [55], ISO/TS 21623 [56], ISO/TS 21361:2019 [57] and by the European Committee for Standardization (CEN) CEN/TR 13205 [58]. During the design of the production process, manufacturers should take into account the possible layout of the building (administrative, for storage, etc.) with the aim of limiting possible contamination.

Although is true that for chemicals, chemical hazard assessments can be a complicated process, it is also true that for nanoparticles, like aeroparticles, it is particularly difficult. Nanomaterials have the same chemical composition as their conventionally sized counterparts; but, with a decrease in the particle size, there is a corresponding increase in the surface area per mass. Additionally, they are size-variable materials with different size-effects and, hence, consequences for humans and the environment.

### 2.4. First Aid Measures [47,59]

Manufacturers, workers and all persons exposed to aeroparticles should be invited to follow and apply this set of recommendations, considering that aerogel-based materials, such as silica, hybrids, etc., are hydrophobic so they may cause temporary dryness and/or irritation of the skin, eyes and mucous membranes. Handling may cause dryness and irritation of the skin and eyes. In the case of inhalation or respiration of dust, people involved must be moved to an open environment. To prevent harm to human health, the following rules should be applied [60]:

Mucuos membranes: Inhalation and respiration of dust during handling may cause temporary irritation of the upper respiratory tract. To clean the throat, people need to drink plenty of fresh water and blow their nose. Seek medical assistance if symptoms arise. 

Eyes: Eyes have to be washed with running water for a few minutes. Seek medical attention if irritation persists. 

Skin: Dust may cause abrasion injuries by rubbing. In all cases, mechanical friction must be avoided to prevent skin irritation. Wash the skin with soap and water. Seek medical assistance if irritation occurs. Do not rub the skin. Wash clothes before reuse. 

Ingestion: Although undesirable effects are not expected, avoid ingestion. Contact a doctor.

### 2.5. Fire-Fighting Measures 

Suitable Extinguishing Media: Carbon dioxide (CO_2_), dry powder or water jet. To extinguish large fires, use water jet or foam. Follow the usual fire prevention procedures to avoid inhalation of the fumes and gases produced by the fire.

## 3. Risks Assessment of Nanomaterials

### 3.1. Health Risks

As aerogels have different properties than their bulk compounds, there is less knowledge available as to how they will react in an environment with human contact [37] and it would be reasonable to measure the concentration of aerogel particles for better establishing exposure limits and the right safety equipment. 

Ultrafine nanoparticles (size less than 100 nm) can be inhaled easily, leading to breathing problems, lung inflammation and distal organ involvement [58]. Ultrafine particles of carbon, silica and titanium are known for causing problems like mesothelioma, pulmonary toxicity, inflammation, genotoxicity and neurotoxicity, DNA damage and human embryo development [61,62,63,64]. The dimension of aerogel nanoparticles can increase the probability of entering into the human bloodstream, together with an increased likelihood of electrochemical reactivity [65].

### 3.2. Safety Aspects of Nanomaterials

The main components of occupational health and safety processes are information gathering, hazard assessment, determination of protection measures, review of the effectiveness of measures, and other documentation [45]. Possible ways of being exposed to nanomaterials are inhalation, dermal absorption and ingestion.

Nanomaterials should be kept in a designated area; cleanliness should be taken care of; and protective gear, like gloves or face masks, should be used. Containers should be labelled with the aerogel material to be used, indicating the properties of the materials. Sealed containers containing the material in free or dry form should be opened in a ventilated hood, fumed hood or closed glove box [66]. Anything previously in touch with the aerogel, meaning the container it was placed in, any wet wipes used to clean it, gloves handling it, masks and so on, is considered contaminated and should be disposed of properly [66]. In the case of protective clothing that can be recovered by cleaning, the effects deriving from disinfection and end-of-life disposal need to be considered in terms of the environmental impacts.

The best way to manage surfaces contaminated by aerogel particles is the adoption of wet wiping or, alternatively, the use of a tack roll mop or strippable decontamination agents [66]. HEPA (High-Efficiency particulate air filter) vacuuming should be used for the capture of airborne particles with diameters below 3 µm. 

Before disposal, aerogel materials should be either kept in a labelled waste container or labelled plastic bags and treated as hazardous waste to minimize the aeroparticles’ dispersion into the environment, as well as the worker’s exposure to the particles [67].

Incineration is one of the most common methods of treating aerogel material waste; additionally, it can be used in soil for construction purposes [68].

### 3.3. Life-Cycle Considerations for the Exposure Assessment and Risk Characterization

Due to the potential release of aerogel particles at different stages (manufacturing, transport, use and disposal), the exposure of the worker, consumer and environment to the nanoparticles should be monitored over the entire life-cycle of the products. In the event that aggregates or agglomerates are formed during a life-cycle step, a(n) (eco)toxicological risk analysis should be engaged considering their potential dissolution or disaggregation, even if the risk of aerogels in these forms is not considered relevant. According to EU recommendations for nanomaterials, all people involved in the exposure to aerogels should consider that “Agglomerated or aggregated particles may exhibit the same properties as the unbound particles” [36] consistent with their potential dissolution or disaggregation, and they have to evaluate the risk due to the potential exposure to constituent particles in the size range of 1–100 nm.

Finally, some studies evaluated the resultant products and production methods of aerogels from an environmental perspective concluding with a positive opinion in terms of net energy, greenhouse gas emissions and less solid waste generation when compared with polyurethane-based materials [69]. A case study also evidenced the beneficial impacts deriving from changing the procedure for aerogel preparation, in terms of reduced CO_2_ emissions and pollution in general, as well as in terms of more ecofriendly adopted solvents [70]. Aside from this, the life-cycle consideration cannot neglect the environmental impact deriving from the production of protective masks, HEPA filters, etc.—which are mainly made of polypropylene (PP), polyehtylene therephtalate (PET), glass microfiber, or melt blow glass fiber—due to the high energy consumption, high costs and the elevated emitted pollutes involved in their own production, which tremendously limit their wide and sustainable applications.

Furthermore, the toxicity effects on the environment as a result of incidental or natural dispersion of aeroparticles should be considered, also including possible toxic effects due to the chemical’s release as this can induce a mismatch in ecosystems and cause a loss of biodiversity. Often, these chemicals are able to leach down the soil, seeping into the water table and killing, as a consequence, aquatic animals and vital soil symbionts, indirectly reaching agro- and animal products and entering the food chain, leading to acute or chronic impacts on human health [71]. 

### 3.4. Aerogels Disposal

This compendium has the ambition of becoming a successful guideline for safely managing aerogel products; but, considering the fact that a lack of knowledge on how to dispose of aerogel materials affects the literature, a brief paragraph has been introduced to raise awareness within the aerogel community and regulatory offices on the need to address this important issue. 

Generally, instruction for aerogel disposal refers to silica-based products, adopting chemical, physical or thermal strategies [44]. Specifically, when a thermal degradation process for either the degradation or recycle step is adopted, the negative impact on the environment linked to the CO_2_ emissions should be considered, thus, when possible, this approach should be avoided [72]. Actually, aerogel wastes end up in landfill or in an incinerator, which over time can lead to the release of volatile aeroparticles in the atmosphere, as well as to the deposit of and significant accumulation of dust which over time can affect the performance and safety of disposal plants, with direct and adverse environmental and human health impacts. These remarks should also be extended to bio-based aerogels, which are generally considered to be environmentally friendly and non-harmful because when they are disposed in landfills they can be subjected to breakage, thus emitting dust which negative affects the environment and human health. Furthermore, considering aerogels can potentially contain additives [44], industrial waste landfills should be advised on the right waste procedures to be taken into account.

## 4. Action for Preparedness and Response Plan 

An interdisciplinary action for promptly managing and limiting risk and to ensure health surveillance measurements to safeguard workers and consumers should be managed and based on the following points, according to the existing EU procedure [73]: 

Protective and preventive risks measures must be designed and applied, as well as adequate job training for protecting people from injury.

Training activities on the purpose and use of the aerogel risk governance and risk management strategies to prepare healthcare staff and public health staff, undertaken in close cooperation with the relevant European Union agencies and bodies and professional health organizations. 

Training is also a continuous cycle of planning, organizing, training, equipping, exercising, evaluating and taking corrective action to respond to all hazard incidents and emergencies.

Improved understanding of the needs and the risk perception.

Risk recognition is the first step to avoid and mitigate dramatic implications.

Exposure to risks should be avoided by adopting less toxic, sustainable and biodegradable materials and chemicals, and adhering to proper safety precautions during the application of aerogel-based materials for the purpose of eliminating or reducing the danger of hazards associated with aeroparticles and agglomerates.

Risk communication for hazardous substances when present. Another recommendation is the adoption of the “safety-by-design” principle for eliminating or lowering the hazards of the aerogel nanoparticles.

Where an alert is notified, a risk assessment concerning the latent severity of the threat to public health, including possible public health measures, shall be addressed by one or a group of the European Union agencies or bodies focused on this: the ECDC (European Centre for Disease Prevention and Control), EMA (European Medicine Agency), EFSA (European Food Safety Authority), ECHA (European Chemicals Agency), EEA (European Environment Agency) and EMCDDA (European Monitoring Centre for Drugs and Drug Addiction).

Development of tools to monitor new and emerging risks and to support longer-term activities to deal with these new risks.

## 5. Conclusions

Aerogels are a special class of nanostructured highly porous materials which have an attractive portfolio of emerging applications due to the interesting physicochemical properties, with lightweight, highly specific surface areas and a tunable surface chemistry. Specifically, aerogel technology is rapidly expanding in the field of environmental and biomedical applications considering the great potential of aerogels. The growing interest in aerogel materials comes from most relevant stakeholders including academia, public research institutions, industry, regulatory agencies and environmental organizations. Similarly, to other chemical substances, it is clear that aerogels pose specific challenges which may be adapted to existing testing methods and assessment approaches.

Up to date aerogel materials are improperly considered as nanomaterials and, likewise, are managed with a lack of information regarding the exposure safety limit. In this scenario, the significance of this compendium is to underline the necessity of an alignment of regulatory and Good Laboratory Practices which should be applied on aerogel management. Furthermore, this compendium also aims to bring the aerogel community a guideline for “Workplace safety practice” for the proper handling of aerogels.

It is well known that the continuous monitoring and supervision work of the European Union (EU) guarantees and ensures the upgrade of regulations, methods and protocols to be followed for limiting potential risks for human health, but in the case of aerogel-based materials, other efforts should be conducted in this sense. As long as a better understanding of the dangers associated with the uses and production of aerogels will not be investigated, the authors want to point out the necessity of providing a global regulatory agreement on aerogels guidance for the regulatory testing of these materials in relation to exposure modalities and the potential toxic effects. Therefore, it is necessary to critically evaluate how the properties of aerogels are linked to adverse human health effects. The identification and the application of a common approach and tools for testing and evaluating the biological effects of aerogel particles would be useful for both regulators and industries working on these new materials.

The aerogel field will maintain its quick expansion in the upcoming years. Based on this scenario, all people, from researchers to industrial manufacturers, are strongly encouraged to adopt appropriate risk management strategies after having critically evaluated the risks to maintain healthy and safe living environment. Since the toxic effects of aerogel-based materials depend on their physicochemical properties (such as shape, solubility and coating) and the dose, administration method/route, exposure duration, etc., there is an urgent need to assess and learn the possibility of these parameters of generating adverse human health effects. Consequently, extensive toxicity studies are required, using both in vivo and in vitro assays, as well as unravelling their molecular mechanisms of action, to determine the potential risks linked to these novel materials.

A future perspective for minimizing some negative environmental externalities linked to the supercritical drying method, which is a process based on a large demand of energy, is the use of renewable energy sources to deal with the electricity needed for drying and reducing the processing time. Moreover, a large-scale manufacturing process will generate positive effects on the product and process in terms of environmental performance, applying the concept of scale economy [74].

## Figures and Tables

**Table 1:** Exposure limits and countries.

Country	Exposure Limits
Australia	2 mg/m^3^, TWA ^1^, Respirable
Austria	4 mg/m^3^, TWA ^1^, Inhalable fraction
Finland	5 mg/m^3^
Germany	4 mg/m^3^, TWA ^1^, Inhalable fraction
India	10 mg/m^3^, TWA ^1^
Ireland	2.4 mg/m^3^, TWA ^1^, Respirable dust
Norway	1.5 mg/m^3^, TWA ^1^, Respirable dust
Switzerland	4 mg/m^3^, TWA ^1^
UK	6 mg/m^3^, TWA ^1^, Inhalable fraction
Belgium	10 mg/m^3^, TWA ^1^, Inhalable. 3 mg/m^3^, TWA ^1^, Respirable
China	8 mg/m^3^, TWA ^1^, 10 mg/m^3^, STEL ^7^
France	10 mg/m^3^, TWA ^1^, Inhalable dust. 5 mg/m^3^, Respirable dust
Italy	10 mg/m^3^, TWA ^1^, Inhalable. 3 mg/m^3^, Respirable
Malaysia	10 mg/m^3^, TWA ^1^, Inhalable. 3 mg/m^3^, Respirable
Spain	10 mg/m^3^, VLA, Inhalable. 3 mg/m^3^, VLA ^4^, Respirable
US ACGIH ^2^—TLV ^3^	10 mg/m^3^, TWA ^1^, Inhalable. 3 mg/m^3^, TWA ^1^, Respirable
US OSHA ^5^—PEL ^6^	15 mg/m^3^, TWA ^1^, Total dust 5 mg/m^3^, TWA ^1^, Respirable

^1^ TWA: Time Weighted Average; ^2^ US ACGIH: United States American Conference of Governmental Industrial Hygienists; ^3^ TLV: Threshold Limit Value; ^4^ VLA: Valore Límite Ambientales (Environmental Limit Value); ^5^ US OSHA: United States Occupational Safety and Health Administration; ^6^ Limit PEL: Permissible Exposure Limit; ^7^ STEL: Short Term Exposure Limit TRGS: Technische Regeln für Gefahrstoffe (Technical Rule for Hazardous Materials).

## Data Availability

No new data were reproduced.
